# Supplementary material for: The Use of Star Anise-Cinnamon Essential Oil as an Alternative Antibiotic in Prevention of Salmonella Infections in Yellow Chickens
Source: Antibiotics (Basel). 2022 Nov 9;11(11):1579. doi: 10.3390/antibiotics11111579 (PMC9686846; doi:10.3390/antibiotics11111579)
Supplement: Supplementary file 1 [file antibiotics-11-01579-s001.zip › body weight.pdf]

Table S1. The body weight (BW) of birds in different groups

Number	Group A (challenged-treated)[1]			Group B (challenge-untreated)[2]			Group C (unchallenge-untreated)
	Subgroup A1[3]	Subgroup A2[4]	Subgroup A3[5]	Subgroup B1[3]	Subgroup B2[4]	Subgroup B3[5]	
1	484.6 g	442.2 g	434.6 g	396.6 g	439.4 g	380.2 g	488.2 g
2	417.6 g	495.2 g	500.1 g	407.4 g	378.5 g	437.2 g	514.2 g
3	407.6 g	493.5 g	447.5 g	362.1 g	372 g	430.1 g	486.3 g
4	479.3 g	547.2 g	477.8 g	501.2 g	346.2 g	431.2 g	441.2 g
5	391.2 g	502.3 g	463.5 g	422.5 g	343.5 g	418.2 g	502.9 g
6	407.6 g	453.1 g	429.7 g	348.9 g	364.2 g	323 g	542.9 g
7	551.6 g	429.1 g	427.1 g	253.9 g	311.9 g	429.5 g	488.4 g
8	415.1 g	520.2 g	433.2 g	383.5 g	409.8 g	386.5 g	477.4 g
9	463.9 g	485.1 g	437.8 g	472.1 g	448.6 g	436.3 g	470.7 g
10	478.2 g	548.9 g	439.2 g	295.9 g	451.7 g	382 g	562.2 g
Average BW	449.67 g	491.68 g	449.05 g	384.41 g	386.58 g	405.42 g	497.44 g

[1] Star anise-cinnamon essential oil (SCEO) was supplenmented in the drinking water.

[2] No SCEO was supplenmented in the drinking water.

[3] *Salmonella pullorum* challenged group.

[4] *Salmonella give* challenged group.

[5] *Salmonella kentucky* challenged group.
