# Supplementary material for: The Use of Star Anise-Cinnamon Essential Oil as an Alternative Antibiotic in Prevention of Salmonella Infections in Yellow Chickens
Source: Antibiotics (Basel). 2022 Nov 9;11(11):1579. doi: 10.3390/antibiotics11111579 (PMC9686846; doi:10.3390/antibiotics11111579)
Supplement: Supplementary file 1 [file antibiotics-11-01579-s001.zip › Salmonella isolation rates.pdf]

Table S2. The isolation rates of *Salmonella* in the birds in the *Salmonella pullorum*, *Salmonella give* and *Salmonella kentucky* challenged groups

Challenge strain	Group A (challenged-treated)[1]	Group B (challenge-untreated)[2]
Salmonella pullorum	50%	90%
Salmonella give	40%	80%
Salmonella kentucky	80%	90%

Note: If *Salmonella* is isolated from any samples of cloaca swabs, cecum and mixture organs in a bird, the bird is determined to be a *salmonella* carrier.

[1] Star anise-cinnamon essential oil (SCEO) was supplenmented in the drinking water.

[2] No SCEO was supplenmented in the drinking water.

Table S3. The isolation rates of *Salmonella pullorum* in the bird's different organs in the *Salmonella pullorum* challenged subgroups

Number	Subgroup A1[1]			Subgroup B1[2]		
	Mixture organs[3]	cecum	Cloaca swab	Mixture organs	cecum	Cloaca swab
1	+	-	-	+	-	-
2	+	+	-	-	+	-
3	-	-	-	+	-	-
4	-	+	-	+	-	-
5	-	-	-	+	-	-
6	-	-	-	-	+	-
7	+	+	-	+	+	+
8	+	-	-	+	+	+
9	-	-	-	-	-	-
10	-	-	-	+	+	+
Total	40.0%	30%	0%	70%	50%	30%

[1] Star anise-cinnamon essential oil (SCEO) was supplenmented in the drinking water.

[2] No SCEO was supplenmented in the drinking water.

[+]: *Salmonella* was detected.

[-]: No *Salmonella* was detected.

[3]:Mixture organs refers to the mixture of visceral parenchyma organs including heart, liver and spleen.

Table S4. The isolation rates of *Salmonella give* in the bird's different organs in the *Salmonella give* challenged subgroups

Number	Subgroup A2[1]			Subgroup B2[2]		
	Mixture organs[3]	cecum	Cloaca swab	Mixture organs	cecum	Cloaca swab
1	-	-	-	+	-	-
2	+	-	-	-	+	-
3	-	+	-	-	-	-
4	-	+	-	-	+	-
5	-	-	-	+	-	-
6	-	-	-	-	-	-
7	-	-	-	-	+	+
8	-	-	-	+	-	+
9	-	-	-	+	+	-
10	+	-	-	-	+	-
Total	20%	20%	0%	40%	50%	20%

[1] Star anise-cinnamon essential oil (SCEO) was supplenmented in the drinking water.

[2] No SCEO was supplenmented in the drinking water.

[3]:Mixture organs refers to the mixture of visceral parenchyma organs including heart, liver and spleen.

[+]: *Salmonella* was detected.

[-]: No *Salmonella* was detected.

Table S5. The isolation rates of *Salmonella kentucky* in the bird's different organs in the *Salmonella kentucky* challenged subgroups

Number	Subgroup A3[1]			Subgroup B3[2]		
	Mixture organs[3]	cecum	Cloaca swab	Mixture organs	cecum	Cloaca swab
1	-	+	+	+	+	-
2	+	+	+	+	-	+
3	-	-	+	-	-	-
4	-	-	-	+	-	+
5	-	-	-	-	+	-
6	-	+	+	-	+	-
7	-	+	-	+	-	-
8	-	+	+	+	+	-

9	-	+	+	+	+	+
10	+	+	-	-	+	-
Total	20%	70%	60%	60%	60%	30%

[1] Star anise-cinnamon essential oil (SCEO) was supplenmented in the drinking water.

[2] No SCEO was supplenmented in the drinking water.

[3]:Mixture organs refers to the mixture of visceral parenchyma organs including heart, liver and spleen.

[+]: *Salmonella* was detected.

[-]: No *Salmonella* was detected.
